# Near-care assay of plasma glial fibrillary acid protein and ubiquitin carboxyl-terminal hydrolase isozyme L1 with shorter and prolonged duration exercise

**DOI:** 10.1038/s41598-026-38768-1

**Published:** 2026-02-10

**Authors:** Michael John Stacey, Amanda Barden, Daniel Snape, Barney Wainwright, Iain Parsons, Todd Leckie, Daniel Fitzpatrick, Gerasimos Grivas, Yannis Pitsiladis, Tom Palin, John O’Hara, David Woods

**Affiliations:** 1 Academic Department of Military Medicine, Research & Clinical Innovation, Royal Centre for Defence Medicine, Birmingham Research Park, Vincent Drive, Birmingham, B15 2SQ UK; 2 Department of Surgery and Cancer, Imperial College London, London, UK; 3 Carnegie School of Sport, Leeds Beckett University, Leeds, UK; 4 Department of Intensive Care, University Hospitals Sussex NHS Foundation Trust, Brighton, UK; 5 Institute of Sport, Exercise and Health, University College London Hospitals, London, UK; 6 Physical Education and Sports, Hellenic Naval Academy, Piraeus, Greece; 7 Department of Sport, Physical Education and Health, Hong Kong Baptist University, Kowloon, Hong Kong, China

**Keywords:** Biomarkers, Medical research, Neurology, Neuroscience

## Abstract

Neurobiomarkers measured in peripheral blood can supplement management strategies following traumatic brain injury (TBI). Dual-assay of glial fibrillary acid protein (GFAP) and ubiquitin carboxyl-terminal hydrolase isozyme L1 (UCHL1) is FDA-approved to inform a decision threshold approach (GFAP > 30 µg.L^− 1^ and/or UCHL1 > 360 µg.L^− 1^) for post-TBI neuroimaging. As physical activity and thermal strain often accompany TBI-prone activities, we investigated whether each molecule’s quantification - and, by extension, clinical decisions - could be influenced by exercise-heat stress. In healthy volunteers monitored continuously for body core temperature (Tc), we used the i-STAT Alinity to assess plasma GFAP and UCHL1 responses to exercise in the laboratory (four female, eighteen male trained participants, cycling for 45 min in 32 °C) and field (three female and 22 male recreational marathon runners, finishing time 231 ± 34 min, peak ambient temperature 11 °C). Respective ΔTc overall were 1.42 ± 0.37 °C and 1.87 [1.53, 2.31] °C. With laboratory exercise, GFAP and UCHL1 did not exceed the manufacturer’s decision threshold. Across the marathon, GFAP was stable, whereas UCH-L1 more than doubled (200 [200, 200] vs. 462 [310, 782] µg.L-1, *P* < 0.0001), breaching the decision threshold for neuroimaging in 18/25 runners. Confounding from more severe exercise-heat stress should be considered when interpreting near-care assay of UCHL1 for TBI management.

## Introduction

Traumatic brain injury (TBI) is a disorder with substantial health and economic impacts. In the UK, where TBI is the leading cause of death in the under 40 age group, approximately 2% of the population will present for emergency medical care of head injury each year^1^. Recreational and occupational activities account for 20% of all TBI globally^2,3^, with up to 3.8 million sports-related concussions documented annually in the United States (US)^4–6^. In the US military, one year of deployment on combat operations was shown to associate with a 5% rate of loss of consciousness and 10% rate of altered consciousness^7^, and many of the 413,858 TBI Service Person (SP) episodes of TBI recorded between 2000 and 2019 related to dismounted duties performed in a hot overseas climate^8^.

Quantification of biomarkers indicative of neurological processes (neurobiomarkers) plays an increasing role in the investigation and clinical management of a range of acute and chronic disorders affecting the Central Nervous System (CNS). In TBI, a range of potential clinical applications have been proposed, spanning pre-hospital, emergency, critical care and rehabilitation phases of management^9^. The US National Institutes of Health–National Institute of Neurological Disorders and Stroke (NIH-NINDS) has identified the need for a new approach to characterising TBI more accurately and, in 2022, launched an international initiative with a focus on the acute phase of injury^10^. A biomarker pillar recommending blood-based measures was incorporated in its proposed new framework, alongside a modifier pillar to integrate features influencing clinical presentation and outcome of TBI, including sports-related aetiology.

The year prior, the US Food and Drug Administration (FDA) approved use of a portable point-of-care blood analyser (Abbott i-STAT Alinity) for semi-quantitative measurement of Glial fibrillary acid protein (GFAP) and ubiquitin carboxyl-terminal hydrolase isozyme L1 (UCHL1)^11^. These peptides – reflecting, respectively, astroglial activation and neuronal injury – account for two of the three core neurobiomarkers recommended for acute (< 24 h) post-TBI measurement in the NIH-NINDS policy initiative, based on their diagnostic and prognostic utility. Versus existing technology, their assay on the i-STAT Alinity was found to offer substantial improvements in the time taken to generate results (15 min from sampling), diagnostic accuracy for TBI (specifically, biochemical exclusion of acute traumatic intracranial lesions visualized on computed tomography of the head, in conjunction with other clinical information) and portability (a cartridge based lightweight system, versus the predicate benchtop laboratory instrument). This means that the i-STAT Alinity is the first ‘near care’ blood testing system for use by trained healthcare professionals in concussion and mild TBI. Marketing authorisations for the European Union and United Kingdom quickly followed^12^, and in 2024 the FDA extended approval to whole-blood testing at the point of care for TBI and expanded the sampling window from 12 to 24 h after injury^13^.

The ALERT-TBI study was a key publication informing the 2021 FDA determination, which evaluated the ability of GFAP and UCHL1 to assist physicians in determining neuroimaging requirement for suspected, non-penetrating TBI^14^. Frozen plasma specimens were assayed by chemiluminescent enzyme-linked immunosorbent assay (ELISA), for comparison against pre-specified cut-offs. The Negative Predictive Value (NPV) of the combined ‘decision threshold’ identified by dual GFAP/UCHL1 assay was 99.3% for acute intracranial lesions identified by computed tomography (CT), introducing a potential benefit of up to 40% reduced need for neuroimaging. Subsequently, NPV of 93.3% was demonstrated for the same study population and scenario with the use of GFAP and UCHL1 measured on the i-STAT Alinity cartridge near-care system^15^ - applying slightly adjusted decision threshold values of GFAP < 30 µg.L^− 1^ and UCHL1 < 360 µg.L^− 1^ - with high correlation between i-STAT Alinity and benchtop laboratory equivalent having been demonstrated in the interim^16^. However, only 2.0% of patients contributing samples to ALERT-TBI were categorised as suffering from sports-related TBI.

Among the considerations pertaining to blood biomarker profiling for TBI stratification, confounding factors and extracerebral sources rank highly; it is recognised that, as a large proportion of TBIs are co-incident with athletic or recreational activities, the impact of physical exertion on biomarkers must be investigated further, particularly when markers are known to be expressed in the periphery^17^. Previously we have characterised responses in GFAP and UCHL1, quantified by ELISA, to a cool weather marathon associated with substantial thermal strain^18^. We demonstrated no difference in concentrations, versus rested baseline, for either molecule, in contrast with exercise-associated elevation in serum concentrations of S100 Beta, which is the third of the neurobiomarkers endorsed by NIH-NINDS, and is known to rise with release from extracranial tissues, including muscle^19^. Nevertheless, more recent findings from other forms of physical activity associated with heat stress indicate that GFAP and UCHL1 may vary differentially over time^20,21^, therefore it is important to further interrogate the impact of physiological strain associated with exercise on each neurobiomarker.

Thus, in the present work, we aimed to first explore whether quantification of each molecule by the Alinity iSTAT near-care cartridge system would be feasible for the exposures to exercise-heat stress characterised in our previous work:^17,22^ a matter of particular interest given the more constrained detection ranges provided by the instrument for use in clinical populations. Then, if possible, we further sought to determine pre- to post-responses to these exposures - namely shorter duration laboratory exercise, associated with lower thermal strain (Study 1), and the greater cumulative heat stress of marathon participation (Study 2) - adopting the null hypotheses that GFAP and UCHL1 would be unchanged across each Study.

## Results

Baseline volunteer characteristics are displayed in Table 1.

**Table 1:** Baseline (pre-exercise/-acclimatisation) volunteer characteristics for study 1 (laboratory heat stress Test) and study 2 (field marathon).

	Age, y	Female, *n* (%)	Height, m	Mass, kg	BSA, m^2^
Study 1 (*n* = 20)	30 ± 7	5 (25.0)	177 ± 8	73.3 ± 12.4	1.90 ± 0.18
Study 2 (*n* = 25)	31 ± 5	3 (12))	178 ± 8	75.4 ± 12.0	1.93 ± 0.18
P-value, Study 1 vs. 2	0.4489	0.4347	0.8900	0.6162	0.4102

BSA, Body Surface Area.

Table 2 profiles participant exposures in Study 1 versus Study 2, including physiological responses, and highlights potential differences arising from the respective study populations, exposures and methods of measurement (e.g. for core temperature, use of rectal thermistor in Study 1 vs. Study 2 ingestible telemetry pill). Changes in body mass are uncorrected for fluid intake, urine output or insensible losses.

**Table 2:** Exposure characteristics and physiological responses in study 1 (laboratory heat stress Test) and study 2 (field marathon).

	Study 1	Study 2	*P*-value
Resting Tc, * °C	37.08 [36.91, 37.21]	37.29 [36.98, 37.55]	0.0663
Exercise duration, min	45 ± 0	231 ± 34	< 0.0001
Environmental conditions**	32.0 ± 0.3 °C, 71 ± 4% relative humidity	0.0 °C, rising to maximum 12 °C	N/A
Mean Tc, °C	37.83 {37.71, 37.99]	38.18 [37.73, 38.72]	0.0311
Maximum Tc rise, °C	1.31 [1.18, 1.76]	1.87 [1.53, 2.31]	0.0005
Post-exercise Tc, °C	38.42 [38.33, 38.82]	38.66 [38.16, 39.15]	0.4128
Body mass change (kg)	−1.25 ± 0.27	−1.3 [−0.35, −2.35]	0.7507

*Tc, body core temperature (measured by rectal thermistor in Study 1, by ingestible telemetric pill in Study 2).

**Humidity data lacking for Study 2.

In Study 1, both GFAP and UCHL1 remained below the respective i-STAT Alinity Lower Limit of Quantification (LLOQ) in all sampling (30 µg.L^− 1^ and 200 µg.L^− 1^), such that TBI decision thresholds (also 30 µg.L^− 1^ for GFAP, 360 µg.L^− 1^ for UCHL1) remained unchallenged.

In Study 2, the decision threshold value for GFAP was breached in 1/25 participants, and limited to T24 sampling - i.e. 1/7 participants who attended for next-day measures - with the individual registering plasma GFAP of 34 µg.L^− 1^. All other GFAP values remained < LLOQ, with no significant variation from Baseline (B) to post-marathon (T0) to T24 in the seven participants with next-day results (*P* > 0.9999).

**Fig. 1:**
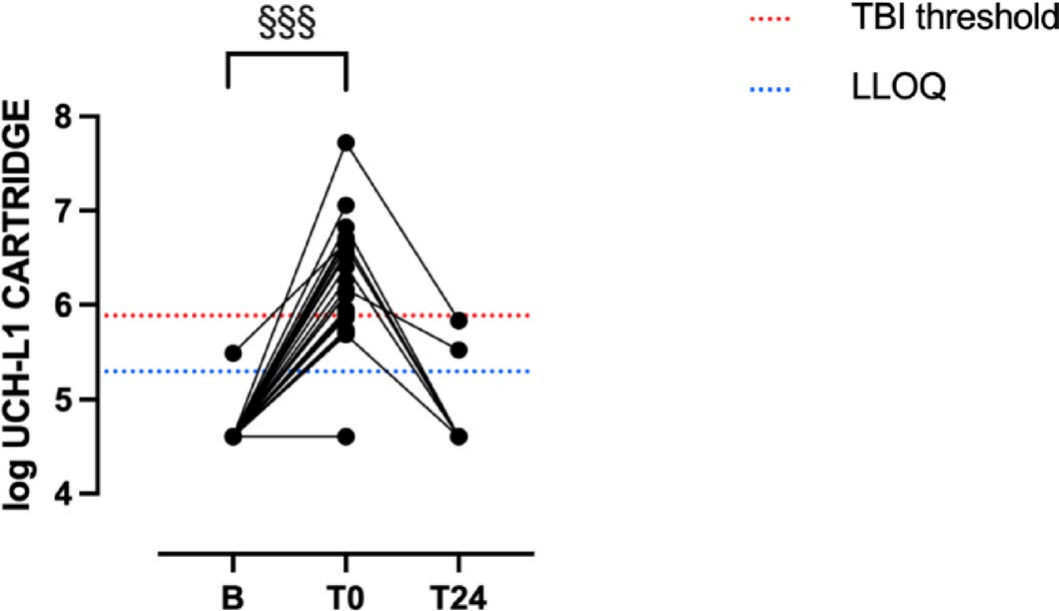
Ubiquitin Carboxyl-terminal Hydrolase isozyme L1 measured by iSTAT Alinity in 25 participants who successfully completed the marathon, sampled at rested baseline (B), upon event completion (T0, *n* = 25) and next-day (T24, *n* = 7). ^§§§^*P* < 0.0001, B vs. T0 (*n* = 25). For *n* = 7 participants with complete B-T0-T24 results, Kruskal Wallis *P* = 0.0021 overall (adjusted *P* = 0.0078 for B vs. T24). LLOQ, lower limit of quantification; TBI, Traumatic Brain Injury (decision threshold). Values below the LLOQ expressed as 50% LLOQ.

In contrast, significant (*P* > 0.0001) increase in UCHL1 from Baseline (B) to post-marathon (T0) meant that 18/25 runners exceeded the corresponding decision threshold for UCHL1 in Study 2 (Fig. 1). In the seven participants with T24 measures, UCHL1 varied significantly (*P* = 0.0021), with T0 values significantly higher than B (adjusted *P* = 0.0078), but no difference between B and T24 (adjusted *P* > 0.9999).

## Discussion

Across two studies distinguished by exercise duration and degree of thermal stress, we have demonstrated differential responses in neurobiomarkers quantified by near-care testing. While i-STAT Alinity measures of GFAP and UCHL1 remained below LLOQ following a shorter, cycle ergometry protocol in the heat (Study 1), and GFAP did not increase with longer duration running (Study 2), significant elevation was observed for UCHL1 in the latter exposure. This was sufficient to breach the manufacturer’s decision threshold for TBI in a majority of participants enrolled in Study 2, whereas no individual exceeded this limit in Study 1.

An urgent need for diagnostic TBI biomarkers has been articulated, not just regarding triage and selection for neuroimaging in the emergency department, but also to support decisions made by medical responders, medics and athletic trainers in the pre-hospital context, as well as to inform critical care and recovery trajectories^9^. The decision-assist function presently offered by the iSTAT Alinity is based on the high sensitivity of elevated GFAP and/or UCH-L1 for acute traumatic intracranial lesions, when interpreted in conjunction with clinical information. The semi-quantitative approach provided by the i-STAT Alinity for measurement of GFAP and UCHL1, whereby both values registering below respective thresholds indicates uncomplicated head injury, presents a rapid alternative to ionising radiation that is less hazardous and potentially less expensive. Although the assay is not approved as a stand-alone diagnostic tool, a positive (elevated) result for either molecule is unlikely to be ignored when selecting patients for brain CT in the initial diagnostic work-up of suspected TBI.

As such, the finding that GFAP was not elevated in either Study 1 or Study 2 should provide reassurance to potential users of the iSTAT Alinity who may be caring for sports-related TBI. This finding was in keeping with our null hypothesis, and was observed despite marathon participation generating substantial cumulative and peak core temperature (Tc), with scope for neuronal stress and blood brain barrier (BBB) opening or disruption in the context of hyperthermia^23^ and repetitive mechanical forces associated with prolonged running. In TBI, the prognostic and predictive power of GFAP - an intermediate filament protein expressed almost exclusively in astrocytes - reflects its temporal elevation some hours distant from CNS insult, passing into the peripheral circulation by a mechanism or mechanisms that remain incompletely elucidated, but likely more reliant on glymphatic and lymphatic transport than the blood-brain barrier^24^.

Considering the diagnostic accuracy of early (< 24 h) measurement of GFAP in complicated TBI, even when this is relatively mild and associated with trauma outside the CNS^25^, it is less likely that additional physical stress, as provided by the present work, would associate with a confounding elevation in peripheral blood titres. While GFAP remained < 30 µg.L^− 1,^ i.e. below its LLOQ, in all but one participant, this is consistent with median plasma concentrations of 15 µg.L^− 1^ assayed on the i-STAT Alinity in healthy individuals unaffected by TBI (95% reference interval 2–51 µg.L^− 1^)^11^, and may reflect values in the present work falling substantially below detectability on the platform. This would be in keeping with reporting of acute GFAP reduction with acute exercise, possibly due to astrocyte activation by cortisol^20^,a hypothesis supported by evidence of robust cortisol secretion with marathon participation in our companion reporting of Study 2^18^. It is also interesting that, across both studies, the solitary GFAP result that did exceed LLOQ arose in a Study 2 participant the day after the marathon, an elevation which is congruent with the temporal profile of GFAP following TBI (detectable at one h, maximally elevated at 20 h). This observation is also in keeping with 16.0% of the healthy volunteers cited above having an elevated plasma i-STAT Alinity result, at rest, of whom all exceeded the decision threshold based on plasma GFAP > 30 µg.L^− 1^ alone i.e. in the presence of a sub-threshold UCHL1^11^.

Consistent findings for GFAP in the present work are in contrast with the divergent responses associated with each exercising exposure for UCHL1. When assayed in the first 24 h following TBI, elevated plasma UCHL1 associates with worse functional outcomes in the longer term, and poorer quality of life^27^. However, even in the absence of TBI, delayed UCHL1 rise has been reported post-exercise, with UCHL1 increasing in proportion to the duration of the bout (aerobic/anaerobic conditioning over 40 to 196 min)^20^, and progressively throughout recovery (immediately post-3 h submaximal cycling, and again at 2 and 24 h)^21^.Both of these preceding protocols employed a benchtop ELISA rather than the iSTAT Alinity; nevertheless, apparent failure of UCHL1 to approach values approximate to the latter’s decision thresholds may have provided relative reassurance to clinical application in the context of prior exercise.

However, in the present work, sampling close to marathon completion revealed relatively greater elevation, with median UCHL1 of 462 µg.L^− 1^ substantially exceeding the iSTAT Alinity decision threshold value of 360 µg.L^− 1^. Factors that may have favoured relative increase in UCHL1 in Study 2, versus Study 1 and previous work, may include the longer time spent exercising, in which UCHL1 could be released from neurons and extracranial sources, such as gonadal tissues>^28^; greater CNS insult from torsional forces increased with running versus static laboratory cycling; relative increase in BBB permeability to enhance UCHL1 entry into the peripheral circulation, with progressive BBB opening observed under exercise at higher core body temperature and with dehydration^19^^[,23^; and impaired UCHL1 clearance by the liver and spleen, due to reduced blood flow with exercise and thermoregulation.

Alternatively, and especially given stability of UCHL1 across the marathon in our allied reporting of Study 2^18^, it is conceivable that the increase we report with marathon participation was artefactual and perhaps related to interfering substances appearing relative to settings of increased physical stress or ingested agents, or to freeze/thawing. On the first point, it is notable that work demonstrating high respective agreement between plasma GFAP and UCHL1 - assayed on the iSTAT Alinity, versus a core laboratory platform - excluded polytrauma cases^22^, a proportion of whom will have been subject to injury affecting multiple other body tissues and associated physiological strain from haemorrhage. Therefore, the sandwich ELISA underpinning the i-STAT Alinity decision threshold may be more sensitive to interference from substances other than GFAP and UCHL1, as regards both detection and capture antibodies, than could be concluded from work published to date. On the second point, no elevation in UCHL1 attributable to assay cross-talk was reported in the FDA approval process with any other tested substance, including caffeine, acetaminophen and non-steroidal anti-inflammatory agents (i.e. common ergogenic aids used by athletes). Thirdly, analysis in the present study was achieved within the post-collection time window recommended by the manufacturer, with the stability of frozen plasma specimens relative to fresh again established as part of the FDA submission process^11,15^.

Considering potential limitations to the generalisability of our work, we acknowledge under-representation of female participants in the study, relative to the general population, though males are more commonly head-injured, with a sex ratio between 2.0:1 and 2.8:1 likely reflecting greater participation of men in high-risk activities that lead to TBIs, including those involving exercise and thermal stress. It does appear that UCHL1 in peripheral blood is slightly higher in males than females, both in TBI and in health^29^; therefore women may not show potential bias to the same degree as men when the Alinity iSTAT is used soon after physical activity. The age of our study cohorts was also relatively young (study 1: 30 ± 7 years; study 2, 31 ± 5 years, Table 1), though again approximately one-half of all patients with mild TBI are between the ages of 15 and 34 years^30^. No participants recruited to either Study 1 or Study 2 were aged < 18 years old, however the iSTAT Alinity is only licensed for use in adults.

Technically, limitations relating to each individual study and our ability to draw direct comparisons between them relate to differences in methods - for measures other than GFAP and UCHL1 assay, which were all made on the iSTAT Alinity – and exclusion criteria. Whereas it was possible to sample for body mass and blood immediately prior to chamber entry in Study 1, access to participants in Study 2 was limited by logistical constraints, meaning that equivalent measurements had to be made the day prior to the marathon. At B, results for GFAP and UCHL1 in Study 2 were all sub-decision threshold values and, where elevated at T0, decreased back below this at T24. This suggests that the acute, prolonged hyperthermic stimulus of the marathon was needed to provoke the responses observed, with the lack of significant rise in either molecule in Study 1 also supporting the idea that values were unlikely to have been elevated by intervening activities pre-marathon.

For similar reasons, body mass calculation and blood collection was potentially delayed by up to 30 min post-exercise completion in Study 2, whereas sampling was conducted immediately upon cessation of 45 min cycling in Study 2. For UCHL1, a proportion of the excursion observed may have related to this additional period of time post-initiation of running, with other investigators accounting for around half the variance in UCHL1 from exercise according to the duration of the bout (between 40 and 196 min)^20^. However, it is highly unlikely that strenuous exercise was undertaken by participants in the relatively narrow post-sampling/pre-marathon window, and under a worst case scenario of all of the participants in Study 2 having been sampled at the maximum 30 min post-event allowable under our protocol, the average time added to the exercise bout itself would only represent 13% of that observed for marathon completion (mean 231 ± 34 min).

Nevertheless, we acknowledge these limitations and the need, in future investigations examining the impact of prolonged exercise-heat stress, for sampling to more tightly bracket the selected exposure. The time gap between B measurements in Study 2 and the actual start of the marathon will have reduced our ability to quantify, accurately, changes in body mass, a limitation compounded by both the withholding of foodstuffs after pre-exercise sampling in Study 1 (not Study 2) and the relative impossibility and unacceptability of measuring nude body mass (NBM) in the field setting of Study 2. Comparing other methods across the two studies, it is likely that Tc estimation in Study 2 was far more likely to be affected by artefactual reduction from cool fluid ingestion affecting measurements from thermistors ingested and still present in the upper gastrointestinal tract, whereas those sited in the rectum in Study 2 will have been relatively protected. However, given the significantly higher Tc recorded in Study 2 than Study 1, it appears that a Type 2 statistical error of this nature was avoided.

Lastly, exclusion criteria including Loss of Consciousness (LOC) from head injury in the preceding four weeks, renal disorders and chronic health complaints were applied to enrolment in Study 2 but not Study 1. As highlighted in the Methods, it is unlikely that these individuals, who remained active in competitive cycling/triathlon outside of study sampling, had been incapacitated by significant TBI or illness in the month prior to participation. Neither did the negative TBI results from Study 1 indicate residual injury or pathophysiology from a CNS insult or extracranial disorder, as has been reported to persist in longitudinal reporting of GFAP in military trauma^31^. All the same, the differing application of exclusion criteria across the studies, and potential differences in the nature of athletic practices pursued by each recruited cohort, may have introduced subtle variation in neurobiomarker response that our design did not control for.

In summary, we have shown that plasma concentrations of GFAP and UCHL1 measured on the Alinity iSTAT appear unaffected by relatively short duration submaximal cycling exercise in hot conditions. However prolonged endurance running, which may associate with significant thermal strain even in cool weather, associates with elevations in UCHL1 that may confound TBI assessment according to the manufacturer’s decision threshold. As the timespan within which diagnostics inform clinical pathways becomes compressed by advancements in available technologies, the accuracy of new methods underpinning decision tools to inform safe patient discharge, versus ongoing monitoring or expedited neuroimaging, becomes more critical. We conclude that caution should be exercised in the clinical interpretation of near- or point-of-care assays where prior exercise-heat stress may be a factor. Further investigation in this area is warranted.

## Methods

### Standard protocol Approvals, Registrations, and patient consents

Two cohorts were recruited to studies of laboratory heat acclimation (Study 1)^22^ and marathon participation (Study 2),^18^ incorporating both recreationally active and more highly trained participants. Individual institutional approvals were granted separately (see Study 1, Snape et al.^22^ and Study 2, Stacey et al.^18^,also featuring complete methods). Favourable opinions were issued from Leeds Beckett University Research Ethics Committee (Study 1, number 84895) and the United Kingdom (UK) Ministry of Defence REC (Study 2, number 1030/MODREC/19). Written informed consent was obtained from all participants in the study. The Declaration of Helsinki, 2024, was respected throughout.

The primary aims of each study (investigating the effects of a mixed-method heat protocol on physiological responses during heat acclimation, and investigating the effects of prolonged endurance running on biochemical surrogates of organ injury, including plasma GFAP and UCHL1 assayed by ELISA) were reported separately and without the inclusion of Alinity iSTAT measures, which were subsequently assayed.

### Study 1 outline

Twenty participants (18 males, four females) underwent a Heat Stress Test (HST) of 45 min fixed-intensity exercise cycle ergometry (Wattbike, Atom X, Nottingham, UK) in an environmental chamber (Sporting Edge UK, Basingstoke, UK) set to target ambient temperature 32.0 °C, target humidity 70%. Core body temperature (Tc) was monitored at 2.5-min intervals. Resting (PRE) and post-HST (POST) blood samples were co-ordinated with body mass measures. Exclusion criteria did not expressly include renal disorders, TBI or ill health (see Study 2 outline below), however all enrolled participants were actively engaged in competitive cycling or triathlon in the lead up to the protocol, with none having recently withdrawn from training or competition due to head injury or decompensated chronic disorders.

### Study 2 outline

Recruitment of 44 entrants at the 2022 Brighton Marathon proceeded at the pre-race exhibition, with inclusion criteria of age 18–55 years and expected finishing time of 4 h or less. Of these individuals, 25 were matched as closely as possible on age and anthropometric grounds to the participants in Study 1. Exclusion criteria additional to those associated with marathon application included congenital renal disease or acquired renal dysfunction requiring medication, brain injury sufficient to cause loss of consciousness in the preceding four weeks, or any other significant chronic health condition (cardiac problems, asthma, diabetes). Exclusion criteria included traumatic loss of consciousness (LOC) i.e. from TBI in the preceding four weeks. Anthropometry measures and blood tests were taken the day prior to the marathon. Body mass was re-measured and further blood tests were taken upon successful marathon completion. Tc was recorded 30s^− 1^. In participants available next-day, blood sampling was repeated.

### Non-biochemical parameters

In study 1, height (Seca, 220, Hamburg, Germany) and body mass (Seca, 770) were measured in preliminary testing, and the latter repeated as towel-dried nude body mass (NBM) both immediately pre- and post-HST. Ad libitum drinking, of water only, was permitted throughout, but no other oral intake was permitted between sampling points. In Study 2, these measurements were repeated, however (i) body mass was sampled in the minimally clothed state, due to limited access to participants and conditions in the outdoor race village (ii) the nature as well as the volume of fluid intake was entirely at the discretion of participants, and (iii) eating, of any foodstuffs desired, was permitted throughout.

In Study 1, Tc was measured by rectal thermistor probe (Variohm, 4492, 400 series, Northamptonshire, UK) connected to a data logger (Grant, SQ2020 2F8, Royston, UK) and inserted approximately 10 cm proximal of the anal sphincter. In Study 2, participants received two e-Celsius ingestible capsules for the measurement of Tc (BodyCap, Caen, France; precision 0.1 °C) and were instructed to swallow the first and second of these 12 and 3 h before the marathon start.

### Blood sampling, processing and analysis

In Study 1, blood was drawn from an antecubital vein after seated rest (*≥* 10 min), just prior to entering the environmental chamber, and likewise immediately post-HST. In Study 2, blood was also collected at the antecubital fossa after 10 min seated rest. In addition to all 25 participants being sampled on the day prior to the marathon (henceforth referred to as Baseline, B0), and within 30 min of passing the finish line (referred to as T0 sampling), in Study 2 blood was collected from *n* = 7 runners who were able to attend for sampling as close to 24 h post-completion as possible (timepoint T24).

In each study, plasma was collected into an EDTA tube (32.332 Sarstedt, Akteingesellscaft & Co., Numbrecht, Germany) and centrifuged within 10 min, for a minimum time of 10 min and at a minimum of 2683 g (i.e. minimum of 26,830 g-minutes; manufacturer recommended minimum g.minutes = 21,000). Aliquots of plasma were stored at − 80 °C Cuntil subsequent analysis, with marathon samples initially held at − 20 °C for a short period, pre-transfer off-site. Dry ice was used for shipping by courier to definitive storage and analysis at Affinity Biomarker Labs (London, UK). Upon thawing, plasma was analysed for GFAP and UCHL1 by Alinity iSTAT (Abbott Laboratories, Illinois, US), with respective lower limit of quantification (LLOQ) of 30 and 200 µg.L^− 11^, upper limits of 10,000 and 3200 µg.L^− 1^, and intra-assay and inter-assay coefficient of variation (CV) of < 10% and < 15%.

### Data handling and statistical analysis

Body surface area was calculated according to the methods of Du Bois & Du Bois^32^. In Study 2, where two thermistors had been ingested and potentially remained in situ throughout the marathon, the higher value was taken to reflect truer Tc, on the basis that ingestion of cooler fluids would decrease measurements where they entered or were adjacent to the intestinal loop containing the e-Celsius capsule. Resting Tc, mean Tc and the maximum rise in Tc (from nadir to peak) were calculated according to this principle, after further curation to interpolate values where artefactual depression was evident in the raw data for < 10 min at a time, or removal of participants’ data entirely where this was exceeded. In all, 6/44 enrolled participants were censored from the study reporting by this mechanism^18^.

Using GraphPad Prism (Version 8.1.0), all data were assessed for normality and expressed as mean ± SD or median [IQR]. Where assay results fell below the LLOQ, a value 50% of the LLOQ was assumed. In order to demonstrate any observable differences between the Study populations and in their respective exposures, comparisons were made by t-test (parametric data) or Wilcoxon test (non-parametric paired data) or Mann-Whitney test (non-paired non-parametric data). Variation in GFAP and UCHL1 in Study 2 was assessed across B-T0-T24 (*n* = 7 participants) by Kruskal-Wallis. Significance was set to alpha = 0.05, with post-hoc correction for multiple comparisons.

## Data Availability

Primary aims of each study were reported separately. Only marathon-derived ELISA measures overlap the present work. Anonymised data not published within the present article will be made available by request from any qualified investigator.

## References

[1] Lawrence, T. et al. Traumatic brain injury in England and wales: prospective audit of epidemiology, complications and standardised mortality. *BMJ Open.* **6**, e012197. 10.1136/bmjopen-2016-012197 (2016). 27884843 10.1136/bmjopen-2016-012197 PMC5168492

[2] Jennett, B. Epidemiology of head injury. *J. Neurol. Neurosurg. Psychiatry*. **60**, 362–369 (1996). 8774396 10.1136/jnnp.60.4.362 PMC1073884

[3] .Guerriero, R. M., Proctor, M. R., Mannix, R. & Meehan, W. P. 3rd. Epidemiology, trends, assessment and management of sport-related concussion in united States high schools. *Curr. Opin. Pediatr.* **24**, 696–701 (2012). 23042252 10.1097/MOP.0b013e3283595175

[4] Kelly, J. P. & Rosenberg, J. H. The development of guidelines for the management of concussion in sports. *J. Head Trauma. Rehabil*. **13**,, 53–65 (1998). 9575257 10.1097/00001199-199804000-00008

[5] Powell, J. W. & Barber-Foss, K. D. Traumatic brain injury in high school athletes. *JAMA* **282**, 958–963 (1999). 10485681 10.1001/jama.282.10.958

[6] Marar, M., McIlvain, N. M., Fields, S. K. & Comstock, R. D. Epidemiology of concussions among united States high school athletes in 20 sports. *Am. J. Sports Med.* **40**, 747–755 (2012). 22287642 10.1177/0363546511435626

[7] Phipps, H. et al. Characteristics and impact of U.S. Military Blast-Related mild traumatic brain injury: A systematic review. *Front. Neurol.* **11**, 559318. 10.3389/fneur.2020.559318 (2020). 33224086 10.3389/fneur.2020.559318 PMC7667277

[8] Hoge, C. W. et al. Mild traumatic brain injury in U.S. Soldiers returning from Iraq. *N Engl. J. Med.* **358**, 453–463 (2008). 18234750 10.1056/NEJMoa072972

[9] Wang, K. K. et al. Blood-based traumatic brain injury biomarkers – Clinical utilities and regulatory pathways in the united States, Europe and Canada. *Expert Rev. Mol. Diagn.* **21**, 1303–1321 (2021). 34783274 10.1080/14737159.2021.2005583

[10] Manley, G. T. et al. A new characterisation of acute traumatic brain injury: the NIH-NINDS TBI classification and nomenclature initiative. *Lancet Neurol.* **24**, 512–523 (2025). 40409315 10.1016/S1474-4422(25)00154-1

[11] United States Food & Drug Administration. – 510(k) Substantial equivalence determination decision summary (2021). https://www.accessdata.fda.gov/cdrh_docs/reviews/K201778.pdf. Accessed 14.12.25.

[12] Abbott Laboratories. https://www.abbott.co.uk/media-center/news/abbott-receives-CE-Mark-for-first-widely-available-lab-based-blood-test-for-traumatic-brain-injury-increasing-access-to-concussion-evaluation.html (2021). )Accessed 14.12.25.

[13] United States Food & Drug Administration. -https://www.accessdata.fda.gov/cdrh_docs/pdf23/K234143.pdf (2024). Accessed 14.12.25.

[14] Bazarian, J. J. et al. Serum GFAP and UCH-L1 for prediction of absence of intracranial injuries on head CT (ALERT-TBI): a multicentre observational study. *Lancet Neurol.* **17**, 782–789 (2018). 30054151 10.1016/S1474-4422(18)30231-X

[15] Bazarian, J. J. et al. Accuracy of a rapid glial fibrillary acidic protein/ubiquitin carboxyl-terminal hydrolase L1 test for the prediction of intracranial injuries on head computed tomography after mild traumatic brain injury. *Acad. Emerg. Med.* **28**, 1308–1317 (2021). 34358399 10.1111/acem.14366 PMC9290667

[16] Korley, F. K. et al. Prognostic value of day-of-injury plasma GFAP and UCH-L1 concentrations for predicting functional recovery after traumatic brain injury in patients from the US TRACK-TBI cohort: an observational cohort study. *Lancet Neurol.* **21**, 803–813 (2022). 35963263 10.1016/S1474-4422(22)00256-3 PMC9462598

[17] Huibregtse, M. E., Bazarian, J. J., Shultz, S. R. & Kawata, K. The biological significance and clinical utility of emerging blood biomarkers for traumatic brain injury. *Neurosci. Biobehav Rev.* **130**, 433–447 (2021). 34474049 10.1016/j.neubiorev.2021.08.029 PMC8511294

[18] Stacey, M. J. et al. Neurobiomarker and body temperature responses to recreational marathon running. *J. Sci. Med. Sport*. **26**, 566–573 (2023). 37777396 10.1016/j.jsams.2023.09.011

[19] Koh, S. X. & Lee, J. K. S100B as a marker for brain damage and blood-brain barrier disruption following exercise. *Sports Med.* **44**, 369–385 (2014). 24194479 10.1007/s40279-013-0119-9

[20] Bazarian, J. J. et al. Effects of physical exertion on early changes in Blood-Based brain biomarkers: implications for the acute point of care diagnosis of concussion. *J. Neurotrauma*. **40**, 693–705 (2023). 36200628 10.1089/neu.2022.0267 PMC10061333

[21] Uddin, N. et al. The effects of exercise, heat-induced hypo-hydration and rehydration on blood-brain-barrier permeability, corticospinal and peripheral excitability. *Eur. J. Appl. Physiol.* **125**, 535–550 (2025). 39340668 10.1007/s00421-024-05616-x PMC11829906

[22] Snape, D. et al. Seven days of mixed-method heat acclimation improved markers of cardiovascular and fluid-regulatory strain during exercise-heat stress. *Exp. Physiol.*10.1113/EP092681 (2025). Online ahead of print. 40864853 10.1113/EP092681 PMC13394165

[23] Watson, P., Black, K. E., Clark, S. C. & Maughan, R. J. Exercise in the heat: effect of fluid ingestion on blood-brain barrier permeability. *Med. Sci. Sports Exerc.* **38**, 2118–2124 (2006). 17146318 10.1249/01.mss.0000235356.31932.0a

[24] Plog, B. A. et al. Biomarkers of traumatic injury are transported from brain to blood via the glymphatic system. *J. Neurosci.* **35**, 518–526 (2025). 10.1523/JNEUROSCI.3742-14.2015 PMC4293408 25589747

[25] Bogoslovsky, T. et al. Increases of plasma levels of glial fibrillary acidic protein, tau, and amyloid β up to 90 days after traumatic brain injury. *J. Neurotrauma*. **34**, 66–73 (2017). 27312416 10.1089/neu.2015.4333 PMC5198034

[26] Zetterberg, H. & Blennow, K. Fluid biomarkers for mild traumatic brain injury and related conditions. *Nat. Rev. Neurol.* **12**, 563–574 (2016). 27632903 10.1038/nrneurol.2016.127

[27] Whitehouse, D. P. et al. Association of Blood-Based biomarkers and 6-Month Patient-Reported outcomes in patients with mild TBI: A CENTER-TBI analysis. *Neurology* **104**, e210040 (2025). 39652812 10.1212/WNL.0000000000210040 PMC11675711

[28] Bishop, P., Rocca, D. & Henley, J. M. Ubiquitin C-terminal hydrolase L1 (UCH-L1): structure, distribution and roles in brain function and dysfunction. *Biochem. J.* **473**, 2453–2462 (2016). 27515257 10.1042/BCJ20160082 PMC4980807

[29] Papa, L. et al. Sex differences in time course and diagnostic accuracy of GFAP and UCH-L1 in trauma patients with mild traumatic brain injury. *Sci. Rep.* **13**, 11833. 10.1038/s41598-023-38804-4 (2023). 37481589 10.1038/s41598-023-38804-4 PMC10363108

[30] Feigin, V. L. et al. Incidence of traumatic brain injury in new zealand: a population-based study. *Lancet Neurol.* **12**, 53–64 (2013). 23177532 10.1016/S1474-4422(12)70262-4

[31] Graham, N. S. et al. Poor long-term outcomes and abnormal neurodegeneration biomarkers after military traumatic brain injury: the ADVANCE study. *J. Neurol. Neurosurg. Psychiatry*. **96**, 105–113 (2025). 39393903 10.1136/jnnp-2024-333777 PMC11877046

[32] Du Bois, D. & Du Bois, E. F. Clinical calorimetry: A formula to estimate the approximate surface area if height and weight be known. *Arch. Int. Med.* **17**, 863–871 (1916). 2520314

